# Association between Experience of Child Abuse and Severity of Drug Addiction Measured by the Addiction Severity Index among Japanese Drug-Dependent Patients

**DOI:** 10.3390/ijerph120302781

**Published:** 2015-03-03

**Authors:** Yasukazu Ogai, Eiichi Senoo, Fumiyuki Chin Gardner, Ayako Haraguchi, Tamaki Saito, Nobuaki Morita, Kazutaka Ikeda

**Affiliations:** 1 Social Psychiatry and Mental Health, Faculty of Medicine, University of Tsukuba, 1-1-1 Tennodai, Tsukuba, Ibaraki 305-8577, Japan; E-Mails: hhd02063@nifty.ne.jp (T.S.); nobuakim@nifty.ne.jp (N.M.); 2 Addictive Substance Project, Tokyo Metropolitan Institute of Medical Science, 2-1-6 Kamikitazawa, Setagaya-ku, Tokyo 156-8506, Japan; E-Mail: ikeda-kz@igakuken.or.jp; 3 Ibaraki Prefectural Medical Center of Psychiatry, 654 Asahi-cho, Kasama, Ibaraki 309-1717, Japan; E-Mail: senoo12001@yahoo.co.jp; 4 College of Medicine, The Pennsylvania State University, 500 University Drive, Hershey, PA 17033, USA; E-Mail: fumiyuki.c.gardner@gmail.com; 5 Hanamizuki Geriatric Health Services Facility, 4-17-1 Jindaiji-Kitamachi, Chofu, Tokyo 182-0011, Japan; E-Mail: mako.haraguchi@nifty.ne.jp

**Keywords:** Addiction Severity Index, drug dependence, child abuse, gender differences, Japanese

## Abstract

The aim of the present study was to clarify the association between child abuse experiences and drug addiction severity among Japanese drug-dependent patients using the Addiction Severity Index-Japanese version (ASI-J). One hundred and eleven inpatients and outpatients with drug dependence participated in the study. Some of the questions on the ASI-J asked about lifetime experiences of abuse. A higher percentage of female participants experienced child abuse compared with male participants. Male participants who experienced child abuse (MEA) had a significantly higher severity of drug use than men who did not experience it (MNEA). Female participants who experienced child abuse (FEA) had significantly more serious problems in family/social relationships than female participants who did not experience it (FNEA). Patients in the MEA group were arrested less frequently for drug charges, experienced more serious problems with their fathers, and experienced more severe anxiety in their lifetime compared with the MNEA group. The FEA group experienced more serious troubles with their sexual partners, close friends, and families and experienced more severe psychiatric problems in their lifetime compared with the FNEA group. These results suggest gender differences in the problems experienced by drug-dependent patients with child abuse experiences, and gender-specific interventions may be more effective in treating their drug dependence.

## 1. Introduction

Drug dependence presents a serious problem in Japan, where methamphetamine is the most commonly used drug by substance abusers. Recently, the abuse of designer drugs, cannabis, and prescription drugs has increased [1]. Drug dependence has caused damage not only to patients but also to Japanese society [2]. For example, approximately 25% of convicted prisoners in Japan have committed offenses under the Stimulant Control Law [3]. Watanabe *et al*. [4] reported that a troubled childhood, such as experiencing serious problems with families, may lead to illegal drug use in adulthood. Sugaya *et al*. [5] reported that patients with drug dependence had difficulty developing good relationships with their families, especially their fathers.

Child abuse victimization is one of the typical problems related to poor family relationships, which is also becoming a severe problem in Japan. The Ministry of Health, Labour and Welfare [6] reported that the number of cases of child abuse in Japan, based on the number of cases treated at child counseling centers in 2012, was approximately 66,000. The incidence of child abuse in Japan was estimated to be approximately 5% among the population under 18 years of age. Morita and Umeno [7] reported that trauma or post-traumatic stress disorder (PTSD) that is caused by experiences of child abuse tended to be complicated by substance abuse disorders.

The association between child abuse and substance abuse has been most reported and discussed in Western countries. In 1990, Brown and Anderson [8] reported that patients who experienced physical abuse in their childhood also experienced drug and alcohol abuse. Locke and Newcomb [9] explained this association using a self-medication theory, in which drug problems may be a response to emotional or psychological distress that stems from experiences of childhood maltreatment. Furthermore, many studies have shown that an abuse experience in childhood is a significant risk factor for substance abuse. For example, a systematic review of studies on child sexual abuse [10], residential patients who were treated for substance abuse [11], detained juveniles who experienced physical and sexual abuse [12], and people who experienced childhood physical and sexual abuse and neglect [13] found that an experience of child abuse (sexual, physical, and psychological) was correlated with or predicted drug or alcohol problems. Some studies identified mediating factors between experiences of abuse and drug/alcohol problems, including PTSD symptoms after sexual abuse [14], anxiety and depression [15], and emotion-based coping [16]. Moreover, gender differences in the relationship between drug abuse and experiences of child abuse were reported. Female substance abusers more frequently experienced physical or sexual abuse during their childhood than male substance abusers [17,18].

The association between child abuse and drug dependence has also been studied in Japanese samples. Fujino and Takahashi [19] distributed a self-report questionnaire to 1,000 inmates who were imprisoned for Stimulant Drug Control Law violations in Japanese correctional institutions. According to their results: (1) females were more likely to be victims of child abuse than males; (2) although the frequency of child abuse victimization was not closely related to criminality, it was closely related to the severity of substance dependence among females, and (3) victims of child abuse tended to be pessimistic and felt that they had no alternative but to rely on illegal substances to cope with social life. In another study, Umeno *et al*. [20] conducted a nationwide cross-sectional survey of 445 drug abusers at drug addiction rehabilitation centers (DARCs) in Japan. They found that: (1) 68% of the participants had experienced some kind of child abuse; (2) female participants more frequently experienced psychological and sexual abuse than male participants, and (3) the abused group had more severe psychological difficulties than the non-abused group.

In summary, studies have shown that child abuse experiences are closely related to adulthood substance abuse. Notably, female substance abusers reported experiences of child abuse more frequently than male substance abusers. These tendencies were also seen among Japanese inmates who were imprisoned for Stimulant Drug Control Law violations and Japanese drug abusers in DARCs. However, insufficient empirical research has been conducted on the relationship between child abuse and drug dependence among Japanese drug-dependent inpatients or outpatients.

### Research Question and Aim

The aim of the present study was twofold. First, we examined the association between child abuse experiences and drug dependence severity among Japanese patients diagnosed with drug dependence or drug abuse. Second, we described the characteristics of drug-dependent patients with histories of child abuse and examined gender differences in these characteristics. We used the Addiction Severity Index-Japanese version (ASI-J) [21], which is a structured interview that measures the severity of multiple problems, including the severity of drug dependence and experiences of child abuse. The present study included information on physical/sexual/psychological child abuse, types of drug used, severity of substance problems, and the severity of other problems in several associated areas, including medical, employment, alcohol, legal, family/social relationship, and psychiatric.

## 2. Methods

### 2.1. Participants and Procedure

One hundred eleven subjects participated in the study, including 41 inpatients, 63 outpatients, and seven non-patients. Eighty-seven, nine, and nine of these participants had a history of drug abuse that primarily involved methamphetamine, cannabis, and methylenedioxymethamphetamine (MDMA), respectively. They were recruited for ongoing research studies at the Tokyo Metropolitan Matsuzawa Hospital, Tokyo (56 subjects), Self Support Services (a recovery facility run by a non-profit organization for addiction recovery), Tokyo (24 subjects), the National Center of Neurology and Psychiatry Musashi Hospital, Kodaira (20 subjects), GAIA (a recovery facility run by a nonprofit organization for addiction recovery), Naha (eight subjects), and Fukko-kai Tarumi Hospital, Kobe (three subjects). The average age of the subjects was 32.9 years (range, 18–60 years; SD = 9.38 years). The participants included 77 males and 33 females and another person whose gender was unknown.

The inclusion criteria were the following: at least 18 years old, a history of drug addiction problems, diagnosed as a drug abuser or drug-dependent based on the criteria of the *Diagnostic and Statistical Manual of Mental Disorders*, 4th edition, inpatient or outpatient at a Japanese mental hospital or recovery facility, non-patient recovering from stimulant abuse in a recovery facility, and ability to understand Japanese. Considering the time required for an interview and reliability of responses, we excluded patients who were in a state of acute drug-induced psychosis. The interviewers included one psychiatrist and four clinical psychologists. The Institutional Review Board of each institution approved the study. All of the participants who provided written informed consent answered the interview and questionnaires.

### 2.2. Measurements

(1) Addiction Severity Index-Japanese version [21]: To measure the severity of drug dependence, the ASI-J, a semi-structured interview that requires approximately 1 h to complete, was administered to the participants. This instrument gathers exhaustive information on seven areas of a patient’s life: medical, employment/support, drug, alcohol, legal, family/social relationships, and psychiatric problems. In each of these areas, various problems that the participants experienced within the last 30 days and in their lifetime, how much they have been bothered by these problems, and how important to them treatment or counseling is for these problems were mainly asked. Severity was rated as a composite score between 0 and 1, calculated based on the patient’s current status.

(2) Child abuse experience: Child abuse in this study was defined as any kind of experience of abuse (psychological, physical, and sexual) inflicted by their families before adulthood (*i.e.*, until 18 years of age). The ASI-J was also used to measure child abuse experiences. This interview included questions about having an experiences of physical abuse, psychological abuse, and sexual abuse in their lifetime. The following questions were asked: “*Did anyone abuse you emotionally (make you feel bad through harsh words)/physically (cause you physical harm)/sexually (force sexual advances or sexual acts) in your life?*” We additionally confirmed who abused them and when these abuses began. Almost every participant who experienced abuse reported that such abuse began in their childhood or adolescence (under 18 years of age) by their parents or older siblings and before they began abusing drugs. Only one participant answered that her sexual partner abused her in adulthood. This participant was regarded and included in the analysis as no child abuse experience.

(3) Stimulant Relapse Risk Scale (SRRS) [22]: The SRRS has 35 items and measures the risk of future drug relapse or drug reuse based on a patient’s behavior and cognition in the last 2 weeks in five dimensions: Anxiety and intention to use drug (e.g., I am anxious about reusing the drug), Emotional problems (e.g., I cannot control my feelings), Compulsivity for drug (e.g., I want to obtain the drug even by working illegally), Positive expectancies and lack of control over drug (e.g., If I use the drug, then I would feel invigorated), and Lack of negative expectancy for the drug (e.g., I would not be able to control myself if I use the drug [reverse coded]). The participants answered each question by selecting one of three choices (1: disagree, 2: neither agree nor disagree, 3: agree).

(4) Visual Analogue Scale (VAS) of subjective craving for drugs: The VAS measured the patients’ subjective desires for a drug by asking the following question: “*Please rate your strongest craving for the drug in the past 2 weeks.*” The participants answered each question by placing a vertical mark on a 100-mm horizontal line, labeled “*not at all*” on the left end and “*extremely*” on the right.

(5) Relapse: To evaluate the risk of relapse, relapse within 3 months after the first rating was investigated. Relapse was operationally defined as the “*use of any drug after the rating*” and judged based on the patients’ self-reports and/or reports from their psychiatrists.

(6) Demographic variables: The participants were also asked to complete a short questionnaire on demographics, including age, sex, and the primary drug they were using (or had used). The question also included the date of their last drug use and period since their last drug use.

### 2.3. Statistical Analysis

The participants were divided into two groups based on having any type of abuse experience in their lifetime. To compare their abuse histories with drug dependence severity by gender, the *χ*^2^ test, Fisher’s exact test, and Student’s *t*-test were applied. Multiple regression analysis was used to examine the influences of child abuse experiences and other variables on the severity of drug abuse by gender. For the analysis of child abuse experience or relapse, “*with child abuse experience*” and “*relapse*” were coded as 1, and “*without child abuse experience*” and “*no relapse*” were coded as 0. All of the analyses were performed using the Statistical Package for the Social Sciences (SPSS) version 18.0 for Windows.

## 3. Results

### 3.1. Gender Differences in Frequency of Abuse Experience

Table 1 shows the frequency of each type of abuse experience by gender. The *χ*^2^ test and Fisher’s exact test showed that experiences of physical abuse, psychological abuse, sexual abuse, and any type of child abuse (coded as experiencing at least one type of child abuse) were significantly associated with gender. These outcomes implied that female participants experienced each or more than one type of child abuse significantly more than male participants.

With regard to the analysis of the relationships between child abuse experiences and the other variables, differences in the type of child abuse experiences were scarcely found. The number of participants who experienced sexual abuse was not sufficiently high to perform a statistical analysis by gender, and no participants had only experienced sexual abuse. Therefore, only the results on the history/experience of child abuse are described below.

### 3.2. Primarily Abused Drugs

The drugs that the participants primarily used and differences in these drugs according to child abuse experience and gender were statistically analyzed. The overlapping counts of the primarily abused drugs revealed that amphetamine accounted for the largest number (72.9%), followed by prescription drugs (11.7%), MDMA (8.1%), cough medicine (8.1%), and cannabis (8.1%). The proportion of multiple-drug abusers was 21.6%. No significant association was found between types of primarily used drugs and abuse experience in either group.

**Table 1:** Gender difference in child abuse experience.

Child Abuse Experience	Male	Female	*p*
History/experience of child abuse			0.026
Experienced	21 (28%)	17 (50%)	
Not experienced	54 (72%)	17 (50%)	
Physical abuse			0.032
Experienced	16 (21%)	14 (41%)	
Not experienced	59 (79%)	20 (59%)	
Psychological abuse			0.001
Experienced	13 (17%)	16 (47%)	
Not experienced	62 (83%)	18 (53%)	
Sexual abuse			0.002
Experienced	0 (0%)	5 (15%)	
Not experienced	75 (100%)	29 (85%)	

The χ^2^ test was used for the analyses, with the exception of sexual abuse, which was analyzed using Fisher’s exact test. The percentage of abuse experience was calculated respectively by gender.

### 3.3. Severity of Drug Addiction

Table 2 shows the gender differences in the association between child abuse experiences and the severity of drug addiction measured with the ASI-J. Male participants who experienced child abuse (MEA) had a significantly higher severity of drug use than those who did not experience child abuse (MNEA). The severity score for alcohol use in the MEA group was lower than in the MNEA group at a marginal level of significance. Female participants who experienced child abuse (FEA) had a significantly higher severity of family/social relationship problems than those who did not experience child abuse (FNEA). The severity score for alcohol use in the FEA group was higher than in the FNEA group at a marginal level of significance.

**Table 2:** Comparison of the composite scores on ASI-J according to child abuse experience and gender.

Composite Score of ASI-J (Range: 0–1)	Male			Female		
	With Child Abuse Experience (*n* = 21)	Without Child Abuse Experience (*n* = 54)	*p*	With Child Abuse Experience (*n* = 17)	Without Child Abuse Experience (*n* = 17)	*p*
Medical status	0.109 (0.24)	0.0548 (0.15)	0.331	0.077 (0.19)	0.107 (0.26)	0.706
Employment/Support status	0.675 (0.24)	0.676 (0.26)	0.993	0.724 (0.21)	0.792 (0.22)	0.365
Alcohol use	0.062 (0.11)	0.129 (0.20)	0.070	0.221 (0.30)	0.054 (0.10)	0.043
Drug use	0.263 (0.20)	0.165 (0.17)	0.033	0.217 (0.20)	0.189 (0.18)	0.678
Legal status	0.017 (0.07)	0.030 (0.10)	0.602	0.044 (0.12)	0.041 (0.13)	0.956
Family/Social relationship	0.229 (0.24)	0.208 (0.20)	0.705	0.418 (0.25)	0.210 (0.20)	0.011
Psychiatric status	0.312 (0.28)	0.255 (0.24)	0.385	0.455 (0.28)	0.308 (0.28)	0.137

The composite score on the ASI-J expresses the severity of the problem in each area between 0 (lowest) and 1 (highest). The mean (standard deviation) was calculated. The data were analyzed using *t*-tests.

Additionally, the influences of child abuse experience and other variables on the severity of drug abuse by gender were examined using multiple regression analysis. The composite score for drug use on the ASI-J was used as the dependent variable. The experience of child abuse, age, and composite scores on the ASI-J in other areas (Medical, Employment/Support, Legal, Family/Social relationships, Psychiatric) were entered simultaneously as the explanatory variables. In males, the experience of child abuse (β = 0.245, *p* = 0.023) and psychiatric status (β = 0.380, *p* = 0.002) significantly influenced the severity of drug abuse. In females, only psychiatric status (β = 0.433, *p* = 0.033) significantly influenced the severity of drug abuse. The influence of the other variables was not significant.

### 3.4. Age, Relapse, Subjective Craving, and Relapse Risk

Gender differences were analyzed in the associations between child abuse experiences and age, relapse within 3 months, subjective craving measured by the VAS, and relapse risk measured by the SRRS. Nineteen of 49 participants for whom information was available relapsed within 3 months. No significant difference was found between these variables, with the exception of the association between child abuse experience and the SRRS subscale “emotional problems” in the male group. The MEA group had significantly higher scores for “emotional problems” than the MNEA group (*t*_44_ = 2.43, *p* < 0.05).

### 3.5. Abuse Experience and ASI-J

We analyzed each item on the ASI-J to evaluate the effects of child abuse experience and gender. Table 3 shows the items for which significant differences were found between the MEA group and MNEA group. In males, the MEA group had significantly lower severity levels on many legal status items than the MNEA group, such as an experience of parole, experience of arrest for a drug charge, number of times charges resulted in a conviction, period of incarceration in lifetime, and period of the last incarceration. With regard to family/social relationships, the score for “*having close relationship with father*” was lower in the MEA group than in the MNEA group. The score for “*serious problems with father in lifetime*” in the MEA group was also significantly lower than in the MNEA group. Additionally, with regard to psychiatric status, the MEA group had a significantly higher level of “*experience of anxiety and tension in lifetime*” than the MNEA group.

Table 4 presents the items in which significant differences were found between the FEA and FNEA groups. In females, the FEA group had significantly higher severity levels on many items related to family/social relationships compared with the FNEA group, including “*living with anyone who has a current drug problem*,” “*experience of serious trouble with sexual partner in lifetime*,” “*experience of serious trouble with close friends in lifetime*,” and “*experience of serious trouble with family in past 30 days*.” 

Additionally, the FEA group had significantly higher severity levels on some items related to psychiatric status than the FNEA group, including “*times of treatment as psychiatric outpatient*,” “*experience of severe depression in lifetime*,” “*experience of hallucinations in lifetime*,” “*experience of serious thought of suicide in lifetime*,” and “*days of psychological problems in past 30 days*.”

**Table 3:** Items on the ASI-J that are affected by child abuse experiences (males).

Items for Which Significant Differences Were Found (Range or Measure)	With Child Abuse Experience	*n*	Without Child Abuse Experience	*n*	*p*
**Legal Status**					
Experience of parole (0 or 1)	0.1 (0.30)	21	0.31 (0.46)	54	0.020
Experience of arrest for drug charge (0 or 1)	0.38 (0.59)	21	1.0 (1.27)	54	0.036
Number of times charges resulted in a conviction	0.43 (0.68)	21	1.39 (1.91)	54	0.002
Period of incarceration in lifetime (months)	1.0 (2.0)	21	15.81 (33.46)	53	0.002
Period of last incarceration (months)	0.89 (1.59)	19	10.06 (18.82)	51	0.001
**Family/Social Relationships**					
Having close relationship with father (0 or 1)	0.1 (0.31)	20	0.55 (0.50)	51	0.000
Experience of serious problems with father in lifetime (0 or 1)	0.86 (0.36)	21	0.51 (0.51)	51	0.002
**Psychiatric Status**					
Experience of anxiety and tension in lifetime (0 or 1)	0.71 (0.46)	21	0.41 (0.49)	54	0.016

For the items that used a binary score between 0 or 1, 0 means “*without experience*”, and 1 means “*with experience*”. For the item “*having close relationship with father*”, 0 means “*no close relationship*”, and 1 means “*having close relationship*”. The mean (standard deviation) was calculated. The data were analyzed using *t*-tests.

**Table 4:** Items on the ASI-J affected by child abuse experiences (females).

Items for Which Significant Differences Were Found (Range or Measure)	With Child Abuse Experience	*n*	Without Child Abuse Experience	*n*	*p*
**Family/Social Relationships**					
Living with anyone who has a current drug problem (0 or 1)	0.24 (0.44)	17	0 (0)	17	0.034
Experience of serious trouble with sexual partner in lifetime (0 or 1)	0.94 (0.24)	17	0.47 (0.51)	17	0.002
Experience of serious trouble with close friends in lifetime (0 or 1)	0.71 (0.47)	17	0.31 (0.49)	17	0.024
Experience of serious trouble with family in past 30 days (0–30)	7.94 (10.70)	17	0.35 (1.00)	17	0.010
**Psychiatric Status**					
Number of times of treatment as psychiatric outpatient	1.00 (1.06)	17	0.31 (0.48)	17	0.024
Experience of severe depression in lifetime (0 or 1)	0.82 (0.39)	17	0.25 (0.45)	17	0.000
Experience of hallucinations in lifetime (0 or 1)	0.71 (0.47)	17	0.29 (0.47)	17	0.016
Experience of serious thought of suicide in lifetime (0 or 1)	0.72 (0.46)	17	0.38 (0.50)	17	0.037
Days of psychological problems in last 30 days (0–30)	17.71 (13.08)	17	6.88 (11.34)	17	0.015

For the items that used a binary score between 0 or 1, 0 means “*without experience*”, and 1 means “*with experience*”. The mean (standard deviation) was calculated. The data were analyzed using *t*-tests.

## 4. Discussion

The present study sought to clarify the associations between child abuse experiences and drug addiction severity among Japanese drug-dependent patients. We found significant gender differences in the association between experiences of child abuse and the severity of drug dependence.

Drug-dependent females experienced more child abuse than drug-dependent males. These results were consistent with the results from other studies that used Japanese drug-dependent subjects [19,20], although our study could not measure the frequency of abuse experience. Additionally, our findings on the percentages of child abuse experience in each gender were similar to the results reported by Fujino and Takahashi, who reported that 30.5% of males and 53.9% of females experienced abuse. Compared with the estimated percentages of child abuse experience in Japan, the incidence of child abuse among drug-dependent patients was very high. Nevertheless, the number of cases of child abuse in Japan is likely to be underestimated because of unreported cases of child abuse that do not receive counseling [23]. Additionally, no significant association was found between child abuse experience and type of primarily used drug, which is similar to the research results with Japanese drug abusers in DARCs [20]. 

The MEA group had a higher severity of drug dependence, more emotional problems, a lower tendency to violate the law, more serious relational problems with their fathers, and more frequent experiences of strong anxiety than the MNEA group. These results indicate that the MEA group had a relatively lower antisocial tendency than the MNEA group. This finding is similar to the findings reported by Fujino *et al*. [19], in which a history of abuse was not significantly related to the severity of crime. However, Logan *et al*. [24] reported that an experience of physical abuse during childhood among males was associated with antisocial behavior in adolescence, such as the perpetration of peer violence. Additionally, the MEA group had relationship problems with their fathers. Considering that they also tended to have emotional problems, this might indicate that their fathers abused them, which may have led to their emotional problems and experiences of anxiety, causing them to progress to drug dependence. Some researchers [25,26] suggested that there are two types of drug abusers whose motivation to use drug is either positive or negative. One type is called a “*sensation-seeker*,” who abuses drugs to seek excitement and satisfy his/her desires. The other type abuses drugs as “*self-medication*” to divert his/her mind from such suffering as negative emotion. The latter is certainly true in many victims of child abuse, which might be the reason for lower antisocial activity in the MEA group compared with other drug abusers, including sensation-seekers, who tend to show a variety of antisocial behaviors, such as delinquency [27]. Considering the finding that the experience of child abuse was significantly associated with the severity of drug abuse in males, these results imply that the MEA group tended to abuse substances more severely to medicate themselves.

The FEA group had a higher severity of family/social relationship problems and alcohol use, more serious relational problems with their sexual partners, close friends, and family members, a tendency to live with drug users, and more frequent experiences of psychiatric problems (severe depression, hallucinations, serious thoughts of suicide, psychiatric treatment, and psychotic problems in the last 30 days) than the FNEA group. These results suggest that experiences of abuse in female drug abusers were mainly related to psychiatric problems, which is consistent with other studies that used Japanese samples [20]. Moreover, compared with male participants, female participants experienced and suffered from various psychiatric problems. Considering the significant influence of the severity of psychiatric problems on the severity of drug abuse, psychiatric problems might have mediated the experience of abuse and severity of drug abuse in the FEA group. Another aspect of the FEA group was their variety of relational problems, especially with their sexual partners. Previous studies found that most female drug abusers’ sexual partners were also drug abusers [28]. Female drug abusers tended to begin using illegal drugs, such as methamphetamine, MDMA, and marijuana, by being introduced to these substances by their sexual partners [29]. To cope with many psychiatric problems that are related to their abuse experiences, the FEA group might have abused different types of substances, such as illegal drugs and alcohol. Morita and Umeno [7] suggested that an increased risk of substance dependence among abuse victims might be attributable to their need to compensate for their suffering from traumatic abuse experiences. Abuse victims may consider using illegal drugs to medicate themselves and ameliorate many types of emotional and mental problems.

One of the limitations of the present study was the sampling procedure. The participants were not randomly recruited and were limited only to inpatients or outpatients who provided informed consent and whose doctors confirmed their ability to answer an interview. Therefore, the data were obtained from cooperative patients with a relatively low severity of drug dependence and not from drug abusers as a whole, such as dropout patients or drug abusers who never sought treatment. Another limitation was the relatively small sample size for an epidemiological study. In particular, the results for the female participants may be underpowered because of their small sample size. The measurement of child abuse experience was an additional limitation. Child abuse victims tend to be resistant to reporting their experience of child abuse [30], and the participants may have been reluctant to answer questions about child abuse in the present study. We conducted retrospective interviews; therefore, the participants’ memories might have been biased. Further research that utilizes larger sample sizes and collects longitudinal and prospective data is needed to confirm the influence of abuse experiences on many problems, including drug use.

## 5. Conclusions

The present study evaluated gender differences in the associations between child abuse experiences and drug dependence among drug-dependent patients. Male drug-dependent patients who experienced child abuse had relationship problems with their fathers and experienced strong anxiety, which might have led to more severe drug use. Female drug-dependent patients who experienced child abuse had relationship problems with their sexual partners, close friends, and families and experienced many types of psychiatric problems. Although both genders appeared to use drugs to cope with their suffering, the aforementioned differences suggest that gender-specific interventions might be useful for maximizing the benefits of interventions.
